# Enhanced Secondary-Electron Detection of Single-Ion Implants in Silicon through Thin SiO_2_ Layers

**DOI:** 10.1021/acs.nanolett.5c05280

**Published:** 2026-01-10

**Authors:** E. B. Schneider, O. G. Lloyd-Willard, K. Stockbridge, M. Ludlow, S. Eserin, L. Antwis, D. C. Cox, R. P. Webb, B. N. Murdin, S. K. Clowes

**Affiliations:** † Surrey Ion Beam Centre, School of Computer Science and Electronic Engineering, 105649 University of Surrey, Guildford GU2 7XH, U.K.; ‡ School of Maths and Physics, 152137 University of Surrey, Guildford GU2 7XH, U.K.; ¶ Ionoptika Ltd., Unit B6, Millbrook Cl, Chandler’s Ford, Eastleigh SO53 4BZ, U.K.

**Keywords:** focused ion beams, single-ion implantation, single-ion detection, quantum technologies, nanofabrication

## Abstract

Deterministic placement of single dopants is essential for scalable quantum devices based on Group V donors in silicon. We demonstrate a nondestructive, high-efficiency method for detecting individual ion implantation events using secondary electrons (SEs) in a focused-ion-beam system. Using low-energy Sb ions implanted into undoped silicon, we achieve up to 98 ± 1% single-ion detection efficiency (DE). We find that introducing thin, controlled SiO_2_ capping layers enhances the SE yield, consistent with the increased electron mean-free path in the oxide, while maintaining successful ion deposition in the underlying silicon substrate. Our approach provides a robust and scalable route to precise donor placement and extends deterministic implantation strategies to a broad range of material systems and quantum device architectures.

## Graphical abstract

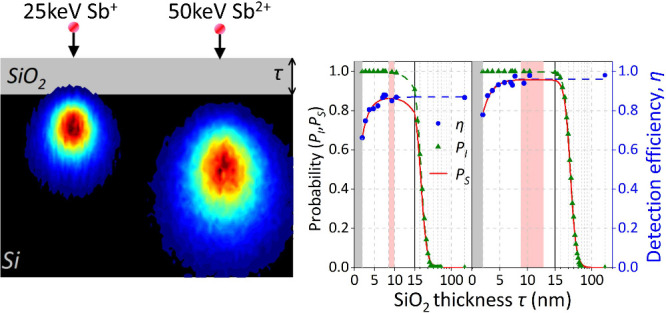

The ability to position and activate individual atoms within a solid with atomic precision represents a defining goal of nanotechnology.^1^ Such control underpins a new generation of quantum devices, in which a single dopant or defect serves as the functional unit. Examples include donor-based spin qubits in silicon,^2–4^ single-electron transistors, and single-photon emitters in wide-band-gap hosts. Ion implantation is a flexible, rapid technique to introduce dopants with a range of species with well-defined energy and spatial localization and is integrated into conventional semiconductor device processes.

For quantum technologies, however, the interest is in single ions. Focused-ion-beam (FIB) tools offer precise control over the position, but determination of the number is also essential. Conventional implantation is inherently stochastic and can be described by a Poisson process. In this case, the probability of implanting exactly one ion per site is limited to 37%, even under ideal conditions. To achieve deterministic placement, each implantation event must therefore be detected in real time, allowing the beam to be promptly terminated.

Current approaches to implant event detection rely primarily on ion-beam-induced current (IBIC) measurements in the target,^5,6^ which demands prefabricated device structures and electrical contacts, thereby restricting throughput and material flexibility. Secondary electron (SE) detection^7–9^ provides a nondestructive, contactless alternative, yet conventional SE-based schemes suffer from lower signal-to-noise ratios and consequently lower event detection efficiency (DE) than IBIC for heavy ions at low energies. High SE efficiency detection has been achieved for many species/host combinations,^9^ but error bars are typically high, and a better understanding of the parameters governing efficiency is needed. Other techniques^10–12^ potentially offer high precision but have not yet demonstrated both high confidence and high throughput.

In this work, we demonstrate high-efficiency detection of single Sb ion implant events into silicon via SE emission, with detection efficiencies up to η = 98 ± 1%. We chose Sb because it is desirable for silicon qubits.^4^ η depends on the SE detector collection efficiency and material SE yield generated by an ion impact. Here we show enhancement of η by adjusting the SE yield through the use of SiO_2_ capping layers that are thin enough that such deposition into the underlying substrate is still highly successful.

The dark count (false positive detection of an ion impact) rate is a factor of 10^–4^ smaller than the ion beam flux, *L*, in our system and can be ignored. In this case, the total number of detected ion implantation events per unit time is *N* = η*L*, where η is the detection efficiency, i.e., the ratio of true positives detected to the total number of true positives. The total number of detected events per pulse is therefore ν = *Nt* = η*Lt*, where *t* is the pulse duration. Assuming that each ion detection event is independent, the detected events follow a Poisson distribution with the mean ν. The probability of an apparently empty pulse is therefore given by^7^

1 $$p_{0}=e^{-\nu }=e^{-\eta Lt}$$

This expression implies that a graph of ν = −ln­(*p*_0_) against λ = *Lt* (the number of ions per pulse) yields a slope equal to that of η. The experiment was designed to obtain *p*_0_(*L*,*t*).

High-resistivity silicon samples were prepared with SiO_2_ layers ranging from 2 to 10.4 nm in thickness and deposited by atomic layer deposition (ALD) using an Ultratech/Cambridge Nanotech Savannah S100 system. The ALD process enables highly uniform oxide growth over a broad thickness range with atomic-layer precision. Deposition was performed on top of the native oxide, and the total SiO_2_ thickness was measured by spectroscopic ellipsometry, which provided subnanometer accuracy in determining the combined oxide thickness. A 200-nm-thick, thermally grown SiO_2_ layer, essentially acting as a bulk substrate, was also measured as a reference.

Ion implantation was carried out using the Single Ion Multi-Species Positioning at Low Energy (SIMPLE) tool (Ionoptika QOne) at the Surrey Ion Beam Centre, with Sb ions implanted at 25 and 50 keV, respectively. These experiments were designed to evaluate the single-ion DE and the yields of secondary particles produced during impact.

For each sample, four arrays of pixels were implanted with pulses having average doses of 0.25, 0.5, 0.75, and 1 ion per pulse. Pixels were spaced by 1 μm to avoid lateral overlap of the implanted ions. During implantation, each pixel was repeatedly pulsed until an above-threshold SE signal was detected by either of two channel electron multiplier (CEM) detectors positioned near the sample. The CEMs were electrostatically biased in a push–pull configuration to collect oppositely charged secondary particles. Once a pixel registered a hit, it was excluded from further pulsing, and the process continued until all pixels in the array produced a detectable event. The number of hits was therefore determined by the array size. The total number of pulses required to make the whole array with one detection per pixel was recorded, and from this, the overall fraction of empty pulses, *p*_0_, was determined.

The uncertainty in *p*_0_ scales with 1/√*n*, where *n* is the total number of pulses used to make the array. To maintain a comparable uncertainty across the different dose conditions, array sizes of 40 × 40, 64 × 64, 70 × 70, and 80 × 80 pixels were used for mean doses of λ = 0.25, 0.5, 0.75, and 1 ion per pulse, respectively. A higher λ means fewer pulses per pixel, so more pixels are needed. With these choices and η ∼ 1, we expect 10,000 pulses per array, and the resulting error in ν ≲ 1% in each case.

The beam current was regularly measured by diverting the beam into a Faraday cup connected to a Keithley picoammeter. This calibration was performed before and after implantation of each set of four arrays, with each run lasting approximately 5 min depending on the array size. The typical current was 220 fA, and so 1 ion per pulse requires *t* = 727 ns. The results for ν­(λ) are shown in Figure 1.

**Figure 1:**
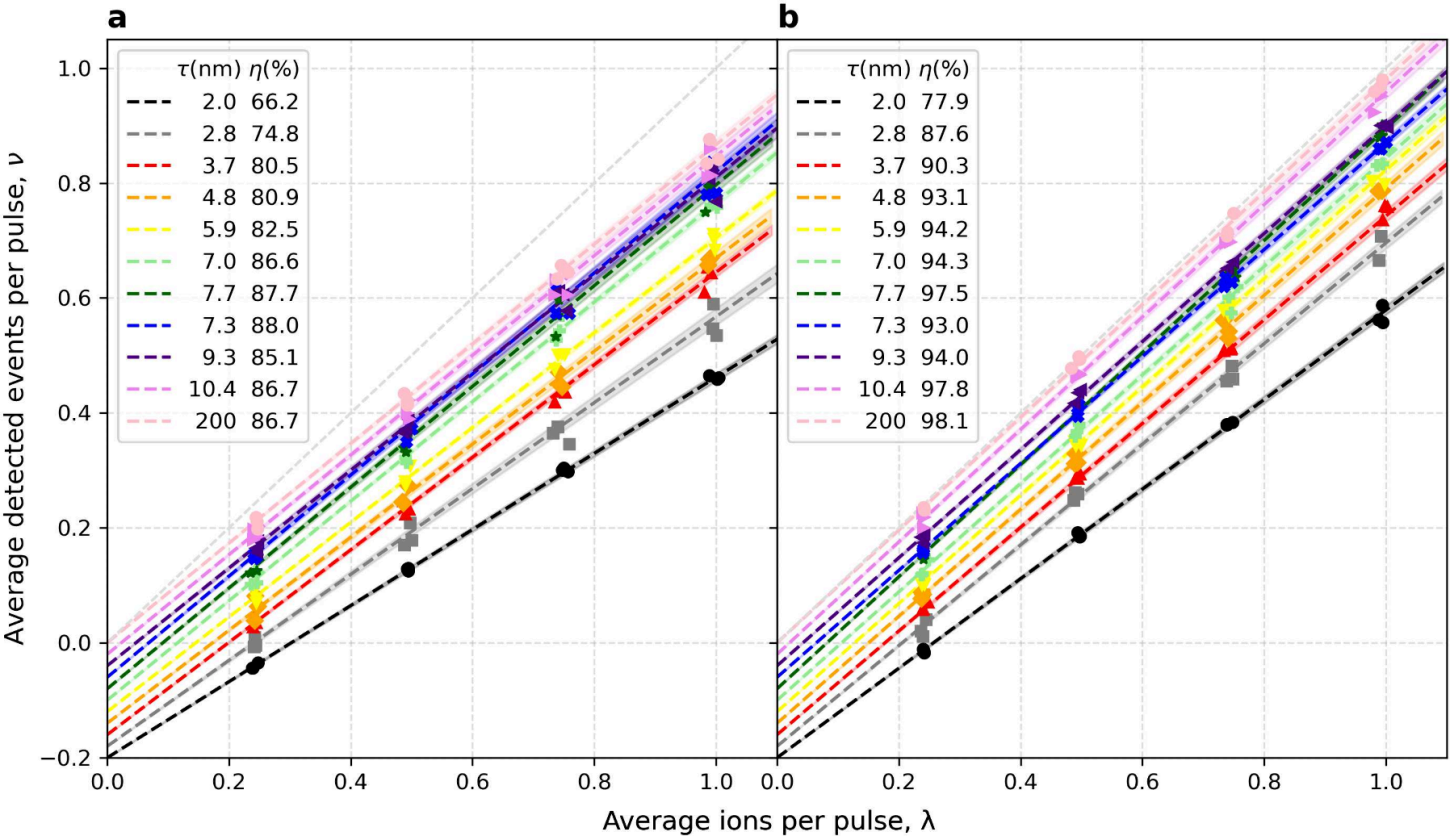
SE DE comparison for varying SiO_2_ capping-layer thicknesses (τ) for (a) 25 keV Sb^+^ and (b) 50 keV Sb^2+^. The plots show experimental ν­(λ) (symbols) for different τ. The slope, η, from linear regression (dashed lines) is indicated in the legend. A line (dashed, light gray) with slope η = 100% is displayed as a reference. The shaded region around each fit line depicts the standard deviation in η. For clarity of display, the intercept from each fit was subtracted off (both the fit and data), and a systematically decreasing vertical offset was then subtracted from each τ data set.

The blanker that defines the ion pulses introduces a latency time, *t*_0_, corresponding to the transit time of ions through the blanker region. Only ions that enter after the blanker opens and exit before it closes reach the sample, effectively shortening the pulse duration by *t*_0_. This produces a nonzero intercept in a graph of ν vs λ but has no effect on the slope.

We ensured that the number of pulses *n* was sufficiently large and that the statistical error in *p*_0_ from the experiment described above was negligible compared to other uncertainties. In contrast, fluctuations in the ion current, *L*, were significant. During a single day of measurement, the standard deviation of the current measured between arrays was typically around 10%, and even though the fluctuation between current calibrations in the experiment was much smaller, this was likely the dominant uncertainty in eq 1. For this reason, to extract η from the data, we performed a standard linear regression using

2 $$T=-\frac{1}{L}\;\ln\left(p_{0}\right)$$

as the dependent variable and the pulse duration *t* as the independent variable. This choice avoids having uncertainty in both axes since fluctuations in the ion current *L* contribute to the experimental error. Physically, *T* = ν/*L* = η*t* can be interpreted as the *effective active detection time* within each pulse: missing impacts due to undetected SEs is equivalent to a detector with a fractional dead time of 1 – η per pulse. We assume that the standard deviation in *T* is constant (and proportional to that of *L*). According to eq 1, the model to be fitted is

3 $$T=\eta \left(t-t_{0}\right)=\eta t+c$$

where η and *c* are free parameters. In short, for presentation purposes, we plot ν = *LT* versus λ = *Lt* in Figure 1, but the regression to obtain η was performed on *T* vs *t*. As seen in Figure 1, the efficiency can be as much as 98%. The error in each η value was also obtained from the regression, and the mean error was 1.0% (with none exceeding 1.5%).

The fit was done separately for each SiO_2_ capping-layer thickness, τ. From the resulting values of the intercept *c*(τ) and slope η­(τ), we obtained values of the blanker latency time *t*_0_(τ). This time depends only on the ion energy and mass and not on the current or details of the sample host. For example, in the case of 25 keV Sb ions, 11 different samples yielded intercepts corresponding to a mean and standard deviation of *t*_0_ = 51 ± 5 ns. This standard deviation is significantly smaller than the rise time of the blanker pulse (10 ns), i.e., consistent with the hypothesis that *t*_0_ is constant.

To determine the probability of a successful implant into the Si substrate through the SiO_2_ capping layer, a distribution of the DE as a function of τ is required. It is apparent from Figure 1 that the slope η increases with the SiO_2_ thickness, τ. Figure 2 shows this trend explicitly. The error bars in the experimental η values, obtained from the regression on Figure 1, are too small to see.

**Figure 2:**
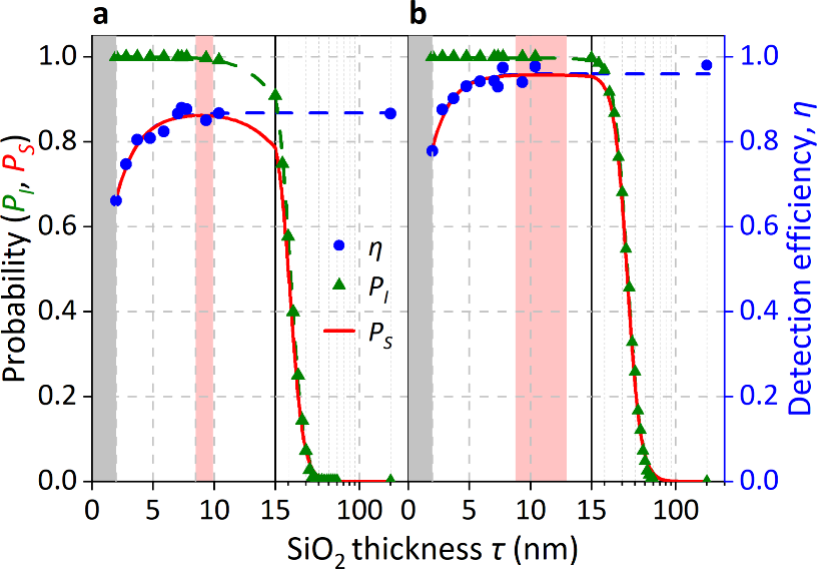
Optimal SiO_2_ capping-layer thickness (τ) determination for maximal implant success probability of (a) 25 keV Sb^+^ and (b) 50 keV Sb^2+^. The SE DE [η­(τ)] data from Figure 1, is shown by the blue points. The error bars at these points are too small to see. The blue dashed line is a guide to the eye of the form *A* – *a* exp­(−τ/τ_0_). The probability of an ion stopping in the Si substrate, *P*_I_(τ), is shown by green triangles. The green line is a guide to the eye of the form 1/[1 + (τ/τ_0_)^*k*^]. The total implant success probability, *P*_S_(τ), is shown as a red line. The gray shaded region indicates the native oxide thickness range, and the red shaded area defines the range of τ that yields a total implant success probability, *P*_S_(τ), within 99.9% of its maximum value. The data are presented using linear (τ ≤ 15 nm) and logarithmic (τ ≥ 15 nm) *x*-axis scales.

η is significantly higher for 50 keV Sb, saturating at ∼98%, compared to ∼87% for 25 keV Sb. This enhancement arises from the greater electronic stopping at higher ion energies, which transfers more energy to near-surface target electrons and increases SE emission. SRIM simulations show that, in the first 5 nm of SiO_2_, a 50 keV Sb ion generates 31 electron–hole pairs, versus only 21 for 25 keV, which directly explains the higher η.

Additionally, it is necessary to determine the probability that an ion of a given energy penetrates the capping layer, *P*_I_(τ). This can be obtained by simulating ion stopping depths using Transport of Ions in Matter (TRIM),^13^ which calculates the interactions of ions with amorphous targets using the Monte Carlo binary collision approximation.

In the TRIM simulations, each ion trajectory was tracked until it came to rest, producing a Monte Carlo distribution of stopping points in three dimensions. The depth of each ion below the surface was recorded and binned to form a 1D histogram of stopping depths. For a given SiO_2_ capping-layer thickness τ, the probability *P*_I_ that an implanted ion penetrates through the oxide and comes to rest in the Si substrate is obtained by counting the fraction of simulated ions that stop deeper than the SiO_2_/Si interface. Repeating this calculation for different oxide thicknesses yields the distribution *P*_I_(τ). The overall probability of a successful implant into the Si substrate is then

4 $$P_{S}\left(\tau \right)=P_{I}\left(\tau \right)\;\eta \left(\tau \right)$$

which allows the optimal capping-layer thickness τ to be determined from the maximum of *P*_S_(τ).

In the TRIM simulations, targets consisted of a 10000-Å-thick Si substrate layer (ρ_Si_ = 2.3212 g cm^–3^) with an amorphous SiO_2_ surface layer (ρ_SiO_2__ = 2.32 g cm^–3^).^13^ Simulated results were obtained for incident ions of 25 keV Sb^+^ and 50 keV Sb^2+^. The SiO_2_ thicknesses in the TRIM models replicated the experimental conditions, and τ was varied from 15 to 60 nm in 2.5 nm increments to generate a smooth *P*_I_(τ) distribution. The results for *P*_I_(τ) and hence *P*_S_(τ) are shown in Figure 2. We find that the peak in the implant success probability is rather broad, which means that it is robust against poor deposition with an uneven thickness. Within the optimal SiO_2_ thickness range (8.5–9.9 nm at 25 keV and 8.8–13.0 nm at 50 keV), there is a <0.3% chance that Sb will stop in the SiO_2_ layer.

TRIM was also used to calculate the depth of Sb in the Si substrate after implantation through the SiO_2_ capping layers. The resulting Sb depth profiles, plotted as a function of the SiO_2_ thickness in Figure 3a,c, show an approximately linear relationship between the implant depth and capping-layer thickness. This simple dependence arises because the Sb stopping distributions in Si and SiO_2_ are very similar: their electronic stopping powers differ only slightly (8.9 vs 7.8 eV Å^–1^ at 25 keV and 12.5 vs 11.1 eV Å^–1^ at 50 keV), so it matters little if the distribution straddles the interface. The similarity in the electronic stopping behavior also implies that the lateral Sb positioning is essentially unaffected by the SiO_2_ capping layers. Parts b and d of Figure 3 show two-dimensional TRIM profiles of the ion stopping locations in the Si substrate after implantation through a 10 nm SiO_2_ layer, which lies within the optimal thickness range. In bulk Si, Sb ions undergo an average lateral displacement of 3.9 ± 5.0 nm at 25 keV and 6.3 ± 8.0 nm at 50 keV. Introducing a 10 nm SiO_2_ capping layer reduces these values only marginally—to 3.7 ± 4.7 and 5.9 ± 7.5 nm, respectively. Thus, increasing the SiO_2_ capping-layer thickness has essentially no effect on the lateral placement of the implanted ions.

**Figure 3:**
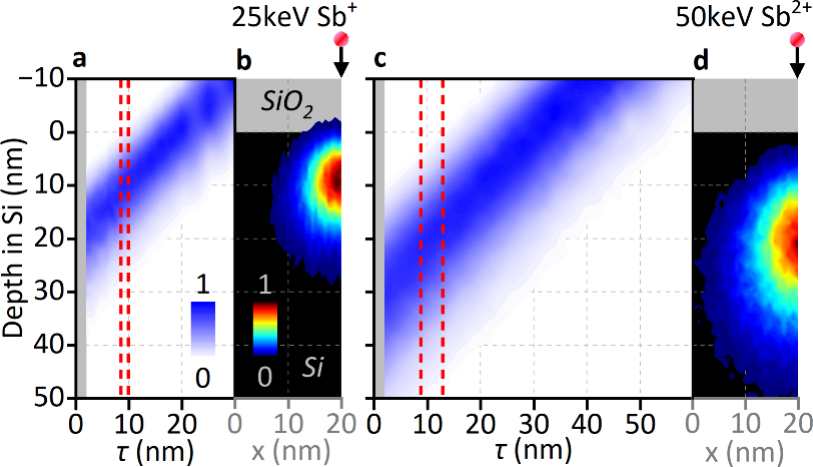
TRIM simulations of Sb in the Si substrate after implantation at (a and b) 25 keV and (c and d) 50 keV through SiO_2_. (a and c) Implanted Sb depth profile as a function of the SiO_2_ thickness (τ), with the color intensity corresponding to the normalized frequency density. The gray-shaded region indicates the native oxide thickness range, and red dashed lines show the optimal τ range determined from Figure 2. (b and d) 2D profiles of the normalized frequency density against the lateral distance (*x*) and depth in Si for a 10 nm SiO_2_ thickness, within the optimal range. The origin of the *y* axis corresponds to where Si interfaces with SiO_2_.

SE detection from a wide range of implant species in both Si and SiO_2_ has been reported by others.^9^ We also consider other available implants here in order to investigate the generality of the improved SE detection using SiO_2_ capping layers and its dependence on atomic mass. Figure 4 shows η as a function of the ion energy: the accelerating voltage was either 8, 12.5, or 25 kV, and experiments were performed with available singly, doubly, or triply charged ions emitted from the source, which were selected using a Wien filter. In Figure 4, we see that η increases with the ion energy, which can be attributed to a corresponding increase in γ.

**Figure 4:**
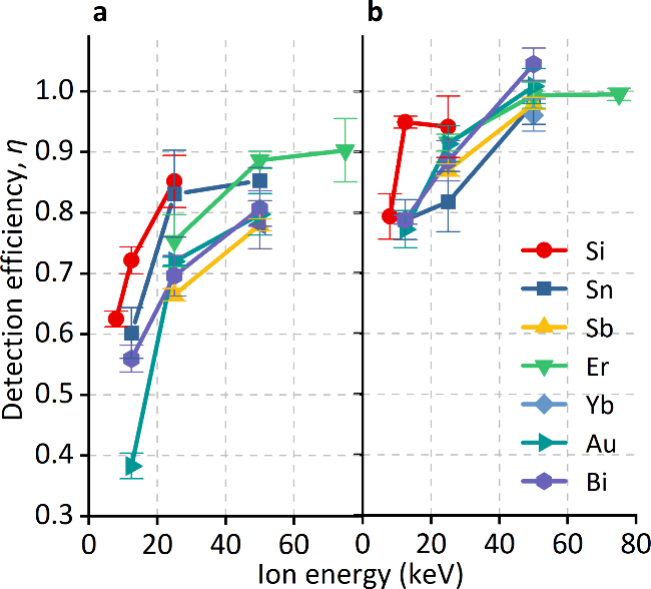
DE as a function of the ion energy for multiple ion species listed in the legend, implanted into (a) Si with native oxide and (b) bulk SiO_2_.

Above, we quantify η from SE, which clearly must increase with the SE yield, γ. Equally clearly, η saturates at large γ values, which makes quantitative conclusions about γ challenging. Nevertheless, we can obtain useful inferences from the trends with ion species, host, and energy.

First, we observe that the saturation occurs when τ = τ_0_ is just a few nanometers, whereas the distribution of ion stopping distances covers a few tens of nanometers. This immediately suggests that the emitted SEs are limited in generation depth by their inelastic mean free path (IMFP), *l*_e_ ∼ τ_0_. If this length scale is significantly less than the ion stopping range, then the material in which the majority of the ions and energy are deposited is irrelevant. This is consistent with Monte Carlo simulation studies, which show that SEs are generated less than about 10 nm from the surface.^14,15^ Therefore, the yield must scale with the energy deposited within *l*_e_ of the surface, *S*_e_ *l*_e_, where *S*_e_ is the electronic stopping power, rather than with the total ion energy *E*:^15^

5 $$\gamma =\frac{Pl_{e}S_{e}}{2J}$$

where *J* is the mean energy required to produce a free electron in the solid and *P* is the mean escape probability for overcoming the surface barrier.

The stopping power *S*_e_ itself depends on the ion species, target composition, and velocity. In our experiments, the ion speeds are well below the Thomas–Fermi velocity (*v* ≪ *v*_TF_ = α*cZ*_1_^2/3^, with α the fine structure constant and *c* the speed of light). In this low-velocity regime, the Lindhard and Scharff (LS) relationship applies,^16,17^ giving

6 $$S_{e}\propto v\frac{Z_{1}Z_{2}}{{\left({Z_{1}}^{2/3}+{Z_{2}}^{2/3}\right)}^{3/2}}$$

where *Z*_1_ and *Z*_2_ are the atomic numbers of the projectile and target, respectively. Our experimental results show enhanced SE DE for faster higher-energy ions of a given species, consistent with this prediction (Figures 1, 2, and 4). Using $v=\sqrt{2E_{1}/A_{1}u}$ , where *E*_1_ and *A*_1_ are the projectile energy and mass and *u* is the atomic mass unit, eq 6 predicts a small decrease (16%) in stopping power from Si to Bi at fixed energy. The modest decline in DE with increasing atomic number observed in Figure 4 is therefore consistent with the LS prediction. A similar slightly decreasing trend of the DE with increasing atomic mass appears to have been observed elsewhere.^9^ We note there is an alternative treatment^17^ of stopping of slow ions with *v* ≪ *v*_TF_ that produces *S*_e_ ∝ *vZ*_1_^2^/*Z*_2_, which is a stronger dependence on species, predicting an order of magnitude increase in yield from Si to Bi, contrary to our observation.

*S*_e_ is the only parameter in eq 5 that depends on other projectile ions, but all of the parameters depend on the target. The LS formula predicts a 29% decrease in stopping power of SiO_2_ compared with Si due to the lower average atomic number *Z*_2_ and 2 for the overall yield. At the same time, the large band gap (*E*_g_ ≈ 9 eV) of SiO_2_ compared with Si (*E*_g_ ≈ 1 eV) suppresses the generation of low-energy electrons by increasing *J*, which also tends to reduce the yield. On the other hand, the IMFP in SiO_2_ is ∼20% longer,^18,19^ and the electron affinity (the energy difference between the conduction band and the vacuum level) is smaller (by about 3.3 eV^20^).

Evidently, the resulting increase in *P* and *l*_e_ for SiO_2_ more than compensates for the decrease in *S*_e_/*J* so that γ increases overall, as observed from Figure 1.

Reported simulations of SE emission under bombardment by focused Ga^+^ ions at 30 keV have found that γ for SiO_2_ was *lower* than that of Si,^14^ while other work reached the opposite conclusion for Ne^+^ in the 1–10 keV range.^15,21^ These conflicts arise from different assumptions about *P* and *l*_e_. Clearly, our observation of a higher yield from SiO_2_ supports the neon studies. However, the neon study^15^ also predicted that, at a fixed ion energy of 10 keV, the SE yield from SiO_2_ increases strongly with projectile mass. This is not consistent with our observation of a slight drop in efficiency as we go from Si to Bi (Figure 4). The contrast between our results and the SE emission modeling^14,15^ is revealing. All cases concern ions of overlapping mass and energy range. In both modeling studies, the electronic stopping and transport processes were treated using semiempirical models whose parameters were optimized for specific materials. Our experimental measurements constrain these parameters.

We expect *P*, *l*_e_, and *J* parameters to be similar for SiO_2_ prepared by ALD or thermal oxidation. Our measurements in Figures 1 and 2 show that η, and therefore γ, of the thermally grown “bulk” SiO_2_ layer is comparable to the native (thermal) oxide plus ALD layers at maximum *P*_S_. This indicates that the deposited and thermally grown oxides have the same composition and that differences in η across samples arise solely from variations in SiO_2_ thickness.

In summary, we have demonstrated a robust, high-efficiency, and nondestructive approach for detecting single-ion implantation events in silicon using SE emission within a FIB system. Beyond providing insight into the basic emission mechanism, our findings also suggest a practical route for optimizing single-ion detection. By introducing a thin SiO_2_ capping layer, we achieved detection efficiencies as high as 98%, produced through calibrated ion-current measurements. The method attains nanometer spatial precision without requiring electrical contacts or prefabricated device structures. Because such oxides can be readily removed by standard chemical etching (e.g., dilute HF), this approach could be extended to a wide variety of target substrates, including semiconductors, metals, and dielectrics, enabling deterministic ion implantation with high detection fidelity in a broad range of materials and species relevant to quantum device fabrication.

## Data Availability

The data supporting the findings of this study are available via Zenodo at 10.5281/zenodo.17804196.
